# Death Literacy and End-of-Life Preparedness Among Health Professions Students: A Mixed-Methods Study

**DOI:** 10.7759/cureus.105767

**Published:** 2026-03-24

**Authors:** Robin J Jacobs, Joshua Caballero

**Affiliations:** 1 Dr. Kiran C. Patel College of Osteopathic Medicine, Nova Southeastern University, Fort Lauderdale, USA; 2 College of Pharmacy, University of Georgia, Athens, USA

**Keywords:** communication, death literacy, end-of-life care, health professions education, interprofessional education, mixed-methods research, palliative care

## Abstract

Background: Health professions students frequently encounter death and dying in clinical settings, yet many report limited preparedness for end-of-life care. Death literacy offers a multidimensional framework for understanding knowledge, experiential learning, communication competence, and navigating systems related to death and bereavement. The objective of this study was to examine predictors of death literacy among health professions students and evaluate differences across program types.

Methods: A cross-sectional, convergent mixed-methods survey was conducted among health professions students at the University of Georgia and Nova Southeastern University. Quantitative measures included the Death Literacy Index-9 (DLI-9) and single-item predictors assessing formal education, communication comfort, experiential exposure to death, perceived program preparedness, and awareness of community resources. Multivariate linear regression identified independent predictors of death literacy. One-way analysis of variance (ANOVA) was used to examine differences by program. Open-ended responses were analyzed thematically and integrated at interpretation.

Results: A total of 326 completed surveys were analyzed. The mean DLI-9 score was 47.0 (*SD* = 18.9). The regression model was significant (*F*(5,312) = 59.25, *p* < .001), explaining 48.7% of variance in death literacy. Greater comfort discussing death was the strongest predictor (β = .435, *p* < .001), followed by awareness of community resources (β = .226, *p* < .001), formal education (β = .147, *p* < .001), perceived preparedness (β = .145, p = .001), and personal experience with death (β = .105, *p* = .011). Death literacy differed significantly by program (*p* < .001), with pharmacy students reporting lower scores. Qualitative findings highlighted the perceived importance of death literacy alongside gaps in formal training and experiential learning.

Conclusions: Communication comfort, educational exposure, and systems awareness are central predictors of death literacy among health professions students. Interprofessional, experiential curricula may strengthen end-of-life preparedness across disciplines.

## Introduction

End-of-life care and health professions education

Health professions students are increasingly expected to provide competent, compassionate, and culturally responsive care to patients and families at the end of life. As populations age and the prevalence of chronic and life-limiting illness rises, encounters with death and dying have become common across healthcare disciplines, not solely within palliative or hospice specialties [1,2]. Despite this, some reports have indicated that students and early-career clinicians feel inadequately prepared to manage the emotional, communicative, and practical challenges of end-of-life care, even while recognizing its importance to professional practice [3-5]. Deficits in readiness have been linked to discomfort with communication, emotional distress, and avoidance of difficult conversations, underscoring the need for improved educational approaches [3,5-7].

Death literacy as a conceptual framework

Death literacy has emerged as a useful framework for understanding how individuals acquire the knowledge, skills, experiential learning, and community awareness needed to effectively engage with death, dying, and bereavement [8]. Unlike constructs such as death anxiety, death literacy emphasizes practical competence, system navigation, and social support in addition to emotional readiness. Within healthcare contexts, death literacy offers a multidimensional lens through which preparedness for end-of-life care can be examined across professions and training stages [8,9].

Measurement of death literacy

The Death Literacy Index (DLI) operationalizes this construct across domains, including personal ability, emotional preparedness, system navigation, and awareness of community resources [10]. Psychometric evaluation of the revised instrument (DLI-R) demonstrates strong construct validity with good model fit in confirmatory factor analysis (CFI = 0.93, TLI = 0.93, RMSEA = 0.06, SRMR = 0.06) and high internal consistency across subscales (Cronbach’s α > 0.80) [9]. The nine-item short form (DLI-9) also shows strong reliability (Cronbach’s α = 0.879; factor α = 0.820-0.872) and excellent agreement with the full index (ICC = 0.987), supporting its validity as a concise measure for community and applied research settings [9].

Death literacy competencies

Formal Education and Training in End-of-Life Care

Formal education in death, dying, and palliative care has been consistently associated with improved knowledge, communication skills, empathy, and confidence among health professions students [3,11,12]. Simulation-based training, interprofessional coursework, and structured curricular exposure have demonstrated measurable gains in perceived preparedness and role clarity in end-of-life contexts across medicine, pharmacy, and allied health disciplines [13-15]. Exposure to end-of-life care during training has also been linked to more positive attitudes and greater knowledge among graduating students [16]. Together, these findings suggest that formal instruction constitutes a foundational component of death literacy development.

Comfort Discussing Death and Dying

Communication competence in discussing death and dying represents a central dimension of clinical readiness. Structured communication training has been shown to improve observable performance in difficult conversations and increase clinician confidence [17]. Educational interventions such as Death Café models, reflective coursework, and required palliative care experiences have further demonstrated increases in student comfort engaging in end-of-life dialogue [5,8,18,19]. Given that communication confidence influences the quality of end-of-life discussions, comfort discussing death may function as a key behavioral expression of death literacy.

Experiential Exposure to Death and Loss

Beyond formal training, experiential exposure, including both clinical encounters and personal bereavement, has been associated with shifts in attitudes, knowledge, and emotional preparedness. Clinical exposure to dying patients has been linked to more positive attitudes and increased knowledge about end-of-life care [16]. Personal experiences with death and loss may deepen emotional insight and professional readiness [20,21]. Pharmacy and medical students with prior bereavement experience demonstrate differences in death acceptance, comfort, and perceived professional roles compared to peers without such exposure [22,23]. These findings suggest that experiential learning contributes to internalized dimensions of death literacy.

Perceived Preparedness and Self-Efficacy

Students’ perceptions of how well their programs prepare them for end-of-life care have been linked to self-efficacy and perceived competence in palliative tasks [24,25]. Educational exposure and experiential learning appear to influence not only objective knowledge but also subjective readiness, which may independently shape engagement in end-of-life care activities.

Rationale for the present study

Although prior research has examined death-related knowledge, attitudes, communication competence, and perceived preparedness within individual disciplines such as medicine, nursing, pharmacy, and allied health professions [3,5,6,17,21,24-27], this work has largely remained discipline-specific. Within medical education, studies document variability in students’ perceived preparedness, communication challenges with dying patients, and the emotional impact of patient death [3,5,6]. Nursing and allied health scholarship has explored trainee attitudes, competence, and educational exposure related to end-of-life care [21,24,25], and pharmacy education studies suggest variability in palliative care curriculum content and limited formal instruction in death and dying topics [26-28].

Despite this growing body of discipline-specific scholarship, comparatively fewer studies have applied an integrated death literacy framework across diverse health professions student populations [8,10]. Evidence suggests that formal end-of-life education improves knowledge and confidence in communicating about death and dying [3,17], experiential exposure contributes to emotional insight and professional readiness [21], and perceived preparedness is associated with self-efficacy in palliative care tasks [25]. The DLI operationalizes this multidimensional construct across domains, including personal ability, experiential knowledge, system navigation, and awareness of community resources [10]. However, limited research has simultaneously examined these predictors within interprofessional student cohorts.

A convergent mixed-methods approach allows for quantitative modeling of predictors while integrating students’ narratives to generate actionable insights for curricular refinement in health professions education [29,30].

Conceptual framework

The conceptual framework guiding this study proposes that death literacy, as operationalized by the DLI, is shaped by four primary predictors: (1) formal education and training in end-of-life care, (2) comfort discussing death and dying, (3) experiential exposure to death (personal and clinical), and (4) perceived preparedness for end-of-life care. These predictors are hypothesized to contribute to multiple DLI domains, including personal ability, emotional preparedness, system navigation knowledge, and awareness of community resources.

Formal education is expected to influence knowledge-based and navigation domains; communication comfort aligns with personal ability and confidence; experiential exposure contributes to emotional preparedness; and perceived preparedness functions as a proximal indicator influencing overall death literacy scores.

Consistent with a convergent mixed-methods design, qualitative student comments are incorporated to contextualize and expand upon quantitative findings. Open-ended responses are used to illuminate students’ interpretations of training experiences, communication challenges, emotional readiness, and perceived curricular gaps. Integration occurs at the interpretation stage, where qualitative themes are compared with quantitative findings to assess convergence, complementarity, or divergence. 

Study aim

Accordingly, the present study examined (1) whether formal education, communication comfort, experiential exposure, perceived preparedness, and awareness of community resources predicted death literacy, and (2) whether death literacy differed across health professions student groups.

## Materials and methods

Study design

A cross-sectional observational design in the form of an online survey was used to examine death literacy among health professions students. A convergent mixed-methods approach was employed, in which quantitative predictors of death literacy were analyzed concurrently with qualitative student responses to contextualize findings.

Approval for this study was granted by Nova Southeastern University’s Institutional Review Board (Protocol #2025-626).

Participants and procedures

The anonymous online questionnaire was distributed via student email listservs to health professions students at the University of Georgia and Nova Southeastern University. Data were collected from November 11, 2025, to February 5, 2026.

Study data were collected and managed using REDCap electronic data capture tools hosted at Nova Southeastern University [31]. REDCap is a secure, web-based data management platform that uses numerical coding to maintain anonymity. Email reminders were sent periodically until study closure.

Survey instrument

The survey included 25 items, comprising demographic and background characteristics (10 research-developed items), Personal Ability (Private Sphere) domain from the DLI-9 (four items), Knowledge and Perceptions (Public Sphere) domain from the DLI-9 (five items), Professional Attitudes and Preparedness (five researcher-developed items), and one open-ended qualitative question.

The demographic section captured participant characteristics and key background variables relevant to death literacy and was developed by the researchers. Items included health professions program, level or year of training, current employment or volunteer work in a healthcare setting, prior formal education or training related to death, dying, or end-of-life care, personal experience with the death of someone close, age, gender identity, race, and Hispanic/Latino/a/x ethnicity.

The Personal Ability (Private Sphere) domain (items taken directly from the DLI-9) comprised four items assessing participants’ perceived ability, confidence, and experiential preparedness related to death and bereavement. These items evaluated respondents’ ability to talk with a grieving person about their loss, assist with feeding or helping a person eat during serious illness or at the end of life, emotional preparedness to support others based on prior experiences of grief or loss, and preparedness to face similar challenges in the future based on those experiences.

The Knowledge and Perceptions (Public Sphere) domain (taken directly from the DLI-9) consisted of five items assessing participants’ knowledge of end-of-life care systems, resources, and navigation. These items evaluated respondents’ knowledge of documents needed when planning for death, ability to navigate the healthcare system for dying patients, knowledge of how to access palliative care services, ability to identify culturally appropriate supports for end-of-life care, and awareness of community supports available for caregivers of dying persons.

The Professional Attitudes and Preparedness domain comprised five researcher-developed items assessing students’ attitudes, confidence, preparedness, and interest in further training related to death and end-of-life care. These items evaluated comfort discussing death or dying with patients, families, or peers; awareness of community supports or resources for caregivers; perceived importance of death literacy for health professionals; perceived preparedness of the current program or training to support end-of-life care; and interest in additional education or training related to death literacy or palliative care.

The survey included one researcher-developed open-ended qualitative item inviting participants to provide a free-text response describing how health professions programs could better prepare students to support dying patients and their families.

Statistical data preparation and analysis

Survey data were analyzed using IBM SPSS Statistics for Windows, version 29.0 (IBM Corp., Armonk, NY, USA) [32].

Descriptive statistics were calculated for all variables. Categorical variables were summarized as frequencies and percentages; continuous variables were reported as means and standard deviations. Distributional assumptions were evaluated using visual inspection and assessment of skewness and kurtosis.

Internal consistency reliability of the instrument in the current study sample was evaluated using Cronbach’s alpha to confirm that the scale maintained acceptable reliability in this population. Internal consistency reliability of the DLI-9 in this sample was acceptable (Cronbach’s α = 0.848). Likert-type items were analyzed as continuous variables, consistent with common practice in medical education research. DLI-9 items were coded and linearly transformed to a 0-1 scale. A composite death literacy score was calculated as the mean of completed items and rescaled to a 0-100 metric.

Bivariate associations were examined using Spearman’s rho correlations. Differences in DLI scores across health professions student groups were assessed using one-way analysis of variance (ANOVA), given potential variation in curricular exposure across disciplines. Although program type was associated with DLI scores in unadjusted analyses, it was not retained in the multivariable model after accounting for formal education, communication comfort, experiential exposure, and perceived preparedness, which were conceptually prioritized predictions. Multivariate linear regression identified independent predictors of death literacy. Multicollinearity diagnostics indicated acceptable variance inflation factors (VIF).

Qualitative analysis and integration

Open-ended responses were analyzed using thematic analysis to identify patterns related to communication confidence, emotional readiness, curricular exposure, and awareness of community supports.

Integration occurred at the interpretation stage, where qualitative themes were compared with quantitative findings to assess convergence, complementarity, or divergence. This approach allowed qualitative narratives to explain statistical associations and inform curricular implications. 

## Results

Quantitative results

Four hundred and seven participants returned the questionnaire. After excluding 81 incomplete cases, 326 completed surveys were retained for the final analysis. While eight of the 326 cases had some missing responses, all of the DLI-9 items were answered.

Sample Characteristics

The participants' ages ranged from 18 to 66 years (M = 27, SD = 7.58). The majority of the participants identified as women (n = 231; 70.9%). Fifty-two (16%) were Hispanic/Latino/a/x, and nearly two-thirds reported being white (n = 202; 62%). Table 1 presents the demographic characteristics of the study sample.

**Table 1:** Characteristics of the Sample (N=326) Note: Percentages may not total 100% due to rounding. Abbreviations: APPEs = Advanced Pharmacy Practice Experiences; Ph.D. = Doctor of Philosophy; M.P.H. = Master of Public Health

Variable	n	%
Gender Identity		
Woman	231	70.9
Man	92	28.2
Non-binary/gender diverse	2	0.6
Other/prefer not to report	1	0.3
Hispanic or Latino/a/x ethnicity	52	16.0
Race		
American Indian or Alaska Native	1	0.3
Asian	54	16.6
Black or African American	29	8.9
Native Hawaiian or Other Pacific Islander	1	0.3
White	202	62.0
Mixed	16	4.9
Other	19	5.8
Prefer not to answer	4	1.2
Program of Study		
Medicine	122	37.4
Pharmacy	140	42.9
Other health professions (e.g., public health, psychology/behavioral health, speech language pathology)	64	19.6
Current level/year of training		
Medical (didactic courses)	100	30.7
Medical (clinical rotations)	21	6.4
Pharmacy (didactic courses)	106	32.5
Pharmacy (clinical rotations/APPEs)	33	10.1
Graduate (e.g., Ph.D., M.P.H)	49	15.0
Medical Resident	1	0.3
Undergraduate	9	2.8
Other	7	2.1
Currently work/volunteer in a healthcare setting		
No	147	45.1
Part time	147	45.1
Full time	32	9.8
Interested in additional education/training related to end-of-life care, palliative care, or death literacy		
Yes	232	71.2
No	12	3.7
Maybe	75	23.0
Personally experienced the death of someone close		
Yes	265	81.3
No	61	18.7

Major Study Variable Statistics

Table 2 reports the summary statistics for the major study variables. The mean score for the item that measured death literacy (DLI-9) was 47.0 (SD = 18.9), with higher scores indicating greater levels of death literacy. Four items used a 5-point Likert response, with higher scores indicating more formal end-of-life education/training, more comfort discussing death, and a stronger feeling that their current program or training has prepared them to support patients and families facing end-of-life issues.

**Table 2:** Descriptive Statistics for Major Study Variables ^a^ SD indicates the standard deviation. ^b^ Range indicates the possible score range for each item For the DLI-9, higher scores indicate higher levels of death literacy. Likert-type items were measured on 5-point scales, with higher scores indicating greater agreement or comfort.

Continuous and Likert-type Single Variables	N	Mean	SD^a^	Range^b^
Death Literacy Index (DLI-9)	326	47.0	18.9	0-100
Comfort with discussing death/dying with patients, families, or peers	319	2.99	1.11	1-5
Current program or training has prepared them to support patients and families facing end-of-life issues	319	2.01	1.31	1-5

Frequencies for dichotomous study variables are presented in Table 3.

**Table 3:** Dichotomous Study Variables

Dichotomous Variables	N	Yes n (%)
Awareness of supports for those caring for a dying person	318	97 (29.8)
Formal education/training related to end-of-life care	326	91 (27.9)
Personally experienced the death of someone close	326	265 (81.3)

Bivariate Correlations

Table 4 reports the statistically significant Spearman’s rho correlations between the major study variables. Only those predictor variables that were statistically significantly correlated with the predicted variable were entered into the regression model.

**Table 4:** Spearman Correlations Among Study Variables Values are Spearman’s rho correlation coefficients. Two-tailed significance tests were used. ** p < .01. Sample size ranged from N = 318–326 due to missing data.

Variable	1	2	3	4	5	6
1. DLI-9 Scale	—					
2. Formal education/training	.34**	—				
3. Comfort discussing death	.58**	.28**	—			
4. Awareness of resources	.41**	.21**	.31**	—		
5. Program preparedness	.36**	.26**	.28**	.18**	—	
6. Personal death experience	.23**	.01	.16**	.09	.07	—

Differences in Death Literacy by Program Type

Differences in death literacy scores by program of study were examined using one-way ANOVA. Mean DLI-9 scores differed significantly by program of study. The ANOVA revealed a significant effect of program on death literacy scores, F(2, 323) = 17.53, p < .001. Assumption testing indicated homogeneity of variances (Levene’s test, p = .158).

Post hoc Tukey HSD comparisons demonstrated that students in pharmacy programs had significantly lower death literacy scores (M = 40.3, SD = 16.7) than students in medicine (M = 50.4, SD = 17.8; p < .001) and other health professions programs (M = 54.5, SD = 21.0; p < .001). No significant difference in death literacy scores was observed between medicine and other health professions students (p = .296). Program of study accounted for approximately 10% of the variance in death literacy scores (η² = .098).

Although program type was associated with variability in death literacy, subsequent multivariate regression analysis examined whether formal education, communication comfort, experiential exposure, and perceived preparedness independently predicted DLI scores.

Multivariate Predictors of Death Literacy

A multiple linear regression analysis was conducted to examine whether personal experience with the death of someone close, prior formal education or training in end-of-life care, comfort discussing death and dying, awareness of community supports/resources, and perceived program preparedness predicted DLI-9 full-scale scores in the full sample (N = 318 with complete data). Only these variables were shown to be correlated with the predicted variable (death literacy) and were thus entered into the model.

The overall model was statistically significant, F(5, 312) = 59.25, p < .001, explaining approximately 49% of the variance in death literacy scores. (R = .698, R² = .487; adjusted R² = .479). The standard error of the estimate was 0.136, indicating good model fit.

All five predictors made significant independent contributions to death literacy scores. Comfort discussing death or dying was the strongest predictor (β = .435, B = 0.074, SE = 0.008, t = 9.79, p < .001). Awareness of community supports/resources was also a significant predictor (β = .226, B = 0.092, SE = 0.018, t = 5.20, p < .001). Formal education or training in end-of-life care contributed significantly (β = .147, B = 0.061, SE = 0.018, t = 3.33, p < .001), as did perceived program preparedness (β = .145, B = 0.028, SE = 0.008, t = 3.29, p = .001). Personal experience with the death of someone close was also associated with higher DLI-9 scores (β = .105, B = 0.051, SE = 0.020, t = 2.55, p = .011).

Collinearity diagnostics indicated no evidence of multicollinearity (VIF range: 1.027-1.204).

Overall, higher death literacy was most strongly associated with greater comfort discussing death and dying, followed by awareness of caregiving supports/resources, and both formal and perceived educational preparation; personal experience with death also contributed independently to higher DLI-9 scores. The regression analysis is presented in Table 5.

**Table 5:** Multivariate Linear Regression Predicting Death Literacy Dependent variable = Death Literacy Index-9 (DLI-9). Unstandardized coefficients (B) and standardized coefficients (β) are reported. All predictors were entered simultaneously. No evidence of problematic multicollinearity was observed (all tolerance values > .80; all VIFs < 1.25). Model R² = .487, adjusted R² = .479, F(5, 312) = 59.25, p < .001. Abbreviation: VIF = Variance Inflation Factor

Predictors	B	Std. Error (SE)	Beta (β)	t	Significance	Tolerance	VIF
Formal education/training	.061	.018	.147	3.33	< .001	.848	1.179
Comfort discussing death	.074	.008	.435	9.79	< .001	.831	1.204
Awareness of resources	.092	.018	.226	5.20	< .001	.870	1.149
Program preparedness	.028	.008	.145	3.29	.001	.845	1.183
Personal death experience	.051	.020	.105	2.55	.011	.974	1.027

Greater comfort discussing death or dying with patients, families, or peers emerged as the strongest independent predictor of death literacy (β = .435, p < .001). Awareness of community supports or resources for individuals caring for a dying person was also significantly associated with higher DLI-9 scores (β = .226, p < .001), as was perceived preparedness of one’s educational program or training to support patients and families facing end-of-life issues (β = .145, p = .001). Formal education or training related to death, dying, or end-of-life care independently predicted higher death literacy scores (β = .147, p < .001). Personal experience with the death of someone close was also a significant, though comparatively weaker, predictor of death literacy (β = .105, p = .011).

Taken together, the results indicate that although personal experience with death contributes to death literacy, educational exposure, perceived preparedness, awareness of community resources, and particularly comfort with end-of-life conversations exert stronger influences.

Descriptive Perceptions of Importance, Comfort, and Preparedness 

Responses to single-item measures provided additional context for the composite death literacy scores. Responses were dichotomized for descriptive clarity. Most participants rated the development of death literacy as important or very important for health professionals, yet only a small proportion reported feeling prepared to support patients and families facing end-of-life issues. Approximately two-thirds of respondents reported feeling comfortable discussing death or dying with patients, families, or peers, while fewer indicated awareness of community supports or resources for individuals caring for a dying person. A majority of participants also expressed interest in additional education or training related to end-of-life care, palliative care, or death literacy. These findings informed the interpretation of the subsequent qualitative results.

Qualitative results

Three major themes emerged from a thematic analysis of the responses from the item “Please share any thoughts, experiences, or suggestions regarding how health professions programs could better prepare students to support dying patients and their families." Overall, qualitative findings largely converged with quantitative results, highlighting the high perceived importance of death literacy alongside limited preparedness, moderate comfort levels, and gaps in practical knowledge.

Theme 1. Perceived Importance of Death Literacy Paired With Inadequate Training

Across disciplines, participants consistently emphasized that death and end-of-life care are unavoidable aspects of health care, yet reported little to no formal preparation within their programs. Many expressed surprise or disappointment that such a fundamental topic was absent or minimally addressed in their curricula.

“We all die… and all of us will have patients die. As a second-year medical student, I’ve been shocked and disappointed that we haven’t been taught about death at all.”

“Death and grief should be discussed as often as possible. It’s unavoidable and one of the only guarantees of human life.”

These reflections align with quantitative findings indicating that most participants rated death literacy as important, while few felt adequately prepared to support patients and families facing end-of-life issues.

Theme 2. Desire for Experiential Learning to Cultivate Interpersonal Communication Skills

Participants frequently described discomfort with end-of-life conversations and expressed a desire for experiential learning approaches, including simulations, role-playing, and supervised clinical exposure. Many noted that communication skills related to death and dying cannot be developed through lectures alone.

“Mock conversations or simulations on speaking to a patient who is dying, as well as that patient’s family members, would be very beneficial before rotations.”

“End-of-life conversation simulations could be extremely helpful. Learning how to address sadness, anger, confusion, or silence would leave students more confident in those moments.”

These comments help explain the quantitative pattern of moderate comfort discussing death but low overall preparedness, suggesting that students may feel willing to engage but lack structured opportunities to practice these skills.

Theme 3. Limited Practical Knowledge of End-of-Life Resources

Many respondents reported uncertainty about the logistical and systems-based aspects of end-of-life care, including hospice, palliative care services, documentation, insurance implications, and community resources. Several participants noted that any knowledge they had was acquired through personal or work experiences rather than formal education.

“At my level of education, we have not discussed this at all, and I would not have any resources or helpful information to provide patients.”

“I’m still dealing with legal issues years after a family member’s death. I would really appreciate support in increasing my death literacy, not only for patients, but for myself and my family.”

This theme directly supports lower scores observed in practical knowledge components of the DLI-9 and reinforces the need for explicit instruction on navigating end-of-life systems and resources.

Qualitative Synthesis

In combination, the qualitative themes contextualized and strengthened the interpretation of the quantitative findings by demonstrating that while participants recognize the importance of death literacy and express willingness to engage in end-of-life care, they perceive significant gaps in formal training, experiential learning opportunities, and practical knowledge. These themes underscore the need for more intentional, experiential, and interdisciplinary approaches to death literacy education across health professions programs. 

## Discussion

This study examined multidimensional predictors of death literacy among health professions students using a convergent mixed-methods design. Communication comfort surfaced as the strongest independent predictor of death literacy, followed by awareness of community supports and resources, formal end-of-life education, and feeling that their program has prepared them for caring for patients and their families who are dealing with end-of-life issues. Personal experience with the death of someone close contributed modestly but independently to death literacy scores. Although differences in mean death literacy scores were observed across program types (i.e., medicine, pharmacy, other health professions), individual-level educational and perceptual factors wielded stronger predictive influence than program type alone. Collectively, these findings suggest that comfort communicating about end-of-life issues and knowledge of community resources may represent primary components of death literacy development in health professions education.

Interpretation of key predictors

Feeling comfortable discussing death and dying with patients and their families had the strongest influence in the regression model. This finding is consistent with prior reports indicating that structured communication training improves clinician confidence, skill performance, and perceived preparedness in end-of-life discussions [3,17]. Also supporting the findings of this study, targeted palliative care training and experiential learning opportunities have been associated with greater comfort and confidence in difficult conversations, including delivering bad news, discussing prognosis, addressing goals of care, and guiding end-of-life decision-making [11-15]. Participants who reported greater comfort discussing death and dying may be more likely to initiate conversations, respond empathically to families, and manage emotionally complex clinical situations, all of which are fundamental to optimal end-of-life care [17,16]. Educational models such as simulation, reflective coursework, and Death Café-style discussions have also shown noticeable improvements in participants’ willingness to discuss end-of-life care [18-20]. The qualitative findings in the present study reinforce this interpretation, as participants repeatedly emphasized the need for simulated conversations and structured practice opportunities to build confidence in discussing death and dying.

Awareness of community resources and supports for patients and their caregivers was the second strongest predictor of death literacy. This shows that knowing how to navigate healthcare systems is an important part of end-of-life preparedness. While the bulk of educational research has focused on both emotional and communication readiness, there have been fewer studies focused on knowledge of community resources. Examples of community supports include hospice and palliative care services, bereavement counseling and grief support groups, caregiver respite programs, hospital or community-based social work services, faith-based and cultural organizations, advance directive and estate planning assistance, veterans’ and disability benefits programs, meal and transportation services, and funeral planning or after-death arrangements support. Participants’ open-ended comments reflected uncertainty about these logistical aspects of care, supporting the quantitative association between resource awareness and DLI scores. Death literacy, thus, seems to go beyond communication skills and includes knowing how to navigate and use healthcare and community systems effectively.

Prior formal education related to death, dying, or end-of-life care, and feeling that their current program prepared them to support patients and families facing end-of-life issues both independently predicted death literacy. This is consistent with earlier studies showing that formal palliative care training is linked to a deeper understanding, greater comfort discussing end-of-life issues, and feeling more ready to care for patients at the end of life [3,11-15]. Importantly, even after considering how much training participants received, how prepared they felt still made a difference. This suggests that participants’ own sense of whether their training was helpful may influence how capable they become.

Personal experience with death was associated with higher levels of death literacy, but its effect was less than that of the other four predictors (i.e., comfort communicating about death and dying, awareness of community resources, prior formal education related to end-of-life care, and current program preparation to support patients and families). Earlier research suggests that bereavement can shape attitudes toward death and increase emotional readiness [20,23]. Experience with loss due to the death of a loved one may help develop empathy, which may shape how future health care providers talk to and support patients and caregivers during end-of-life care. However, personal experience by itself may not be enough to develop the full range of knowledge and practical skills involved in death literacy. While personal experience with death may increase emotional awareness, it does not automatically teach students how to have difficult conversations, make care decisions, or find and use available resources. The findings of this study suggest that personal experience can offer insight, but formal training and supervised practice are necessary to fully prepare students to care for patients at the end of life.

Differences between programs of study

Pharmacy students had lower mean death literacy scores compared to medicine and other health professions students. Nonetheless, program type was not kept as an independent predictor in the regression model once communication comfort, education, preparedness, and resource awareness were included. This suggests that differences are more likely due to the amount of training and hands-on experience students receive, rather than the specific profession they are studying.

Medical education traditionally includes more direct exposure to serious illness, hospital-based care, and formal training in difficult conversations, such as delivering bad news and discussing goals of care [3,17]. These experiences may help students feel more comfortable and competent in end-of-life situations. Likewise, some allied health programs incorporate clinical placements and coursework focused on holistic care, patient communication, and teamwork, which may also foster the development of death literacy [25,26]. In contrast, pharmacy programs have varied in how much palliative care and end-of-life content they include, and many have offered limited structured training on death and dying [26,27]. Recent studies continue to show gaps in pharmacy education, including limited training in communication skills and coping strategies related to end-of-life care [28]. These reports are consistent with this research and suggest that focused improvements in the curriculum could help address these differences.

Mixed-methods integration

The themes from the open-ended responses closely matched the survey results. Participants said death literacy was very important, but also reported feeling underprepared, which reflects the lower scores on preparedness found in quantitative data results. Many participants asked for more simulation-based training, which could help explain why comfort discussing end-of-life issues was the strongest predictor in the statistical model. Participants also described limited knowledge about hospice services, documentation, and caregiver resources, supporting the finding that awareness of community resources was an important predictor of death literacy. Taken together, these results increase confidence in the findings. Reviewing the survey data and participant comments helped show where training gaps exist. The participant responses did not contradict the quantitative results; rather, they helped explain how more practice communicating and better training about available patient and caregiver resources and supports could improve death literacy.

Educational implications

The results of this study have several implications for health professions education. Because feeling comfortable about discussing death and dying with patients was the strongest predictor of death literacy, it could be argued that programs should focus on giving students structured practice with difficult conversations. Simulation, role-play, standardized patient encounters, and guided debriefing can help students build confidence in discussing prognosis, goals of care, and end-of-life decisions [17]. These methods give students a safe space to practice before using these skills in clinical settings.

Knowing about community and healthcare resources was linked to higher death literacy. Health professions programs may benefit from teaching students about hospice referrals, palliative care consultation, advance care planning, do not resuscitate (DNR) orders and living wills, caregiver support, and bereavement services. Incorporating case-based exercises focused on hospice referral, DNR documentation, advance directives, and interdisciplinary discharge planning may help students turn knowledge into practical skills.

Two factors, having formal education in end-of-life care and feeling that their program has prepared them for end-of-life situations, were linked to higher death literacy. This shows that training includes two related but different parts. Students who reported formal training had higher death literacy scores, which is consistent with earlier research showing that structured palliative care education improves knowledge, communication skills, and confidence [3,11-15]. Organized instruction, whether through lectures, clinical experiences, simulation, or interprofessional learning, appears to strengthen students’ understanding of end-of-life care.

Feeling prepared remained important even after accounting for formal education, suggesting that just receiving instruction is not enough. How prepared students feel may reflect the quality and hands-on nature of their training. That is, formal training may provide information, but feeling prepared may indicate whether students believe they can use that information effectively in real clinical situations.

Pharmacy students reported lower death literacy scores than medical or other health professions students, which suggests a need for stronger and more consistent end-of-life education in pharmacy programs. One possible explanation is that pharmacy training often involves less direct and sustained patient interaction compared to medicine, nursing, or mental health fields, where students more frequently participate in bedside care, family meetings, counseling sessions, and difficult conversations about prognosis or goals of care. Prior research has shown that palliative care content in pharmacy curricula is often limited or inconsistent, including gaps in communication and coping skills training [26-28]. Because pharmacy students may have fewer structured opportunities to practice end-of-life discussions and observe decision-making in clinical settings, they may have less opportunity to build comfort and practical skills. Expanding experiential learning, structured communication training, and interdisciplinary exposure may help strengthen preparedness and reduce differences across disciplines.

Finally, an interprofessional approach may be especially valuable in strengthening death literacy across disciplines. Bringing students from medicine, pharmacy, and other health professions together for shared training experiences may enhance understanding of professional roles, improve collaborative practice, and support more coordinated end-of-life care. Integrating death literacy concepts throughout the curriculum, rather than limiting them to a single course or rotation, may help reinforce knowledge, communication skills, and systems awareness over time. Such longitudinal and interdisciplinary approaches may better prepare future clinicians to provide effective and compassionate care to patients and families facing serious illness and death. This may be necessary since caregivers may rely on pharmacists to pick up prescription medications for their family members who have been discharged home for end-of-life care. 

Limitations

There are several limitations of this study. The relatively small sample (N = 326) was collected from only two universities in the United States (the University of Georgia and Nova Southeastern University). This survey was voluntary, which may have contributed to self-selection bias since students with a greater interest in end-of-life care may be more likely to complete the survey; thus, generalizations about all health professions students cannot be made. Also, because this study used a cross-sectional design (data collected at one point in time), we cannot determine cause and effect. The associations observed do not show whether one factor leads to another, or which came first. Additionally, although the DLI measures several aspects of competence, it does not evaluate how students actually perform in real clinical situations.

## Conclusions

Death literacy among health professions students is most closely associated with how comfortable they feel talking about death and dying, how aware they are of available community resources, and how well their programs prepare them through formal training. Personal experience with death plays a role, but education and hands-on learning opportunities seem to matter more. Differences between programs likely reflect variations in coursework and training rather than differences between the professions themselves. Interprofessional training, practice with difficult conversations, and clearer teaching about available support services may improve students’ readiness to care for patients and their families at the end of life. Because caring for people with serious illness is a routine part of healthcare, strengthening death-related education can help students become both more capable and more compassionate clinicians.
